# Reply to Letter: “Real‐World Clinical Experience With Serum MOG and AQP4 Antibody Testing by Live Versus Fixed Cell‐Based Assay”

**DOI:** 10.1002/acn3.70169

**Published:** 2025-08-08

**Authors:** Yana Said, Eoin P. Flanagan, Elias S. Sotirchos

**Affiliations:** ^1^ Department of Neurology Johns Hopkins University School of Medicine Baltimore Maryland USA; ^2^ Laboratory Medicine and Pathology, Department of Neurology, Center for MS and Autoimmune Neurology Mayo Clinic College of Medicine Rochester Minnesota USA

**Keywords:** cell‐based assay, diagnostic sensitivity, MOG, MOG‐IgG, myelin oligodendrocyte antibody‐associated disease

We thank Vij et al. for reporting their experience with the comparative performance of a live cell‐based assay (LCBA) versus a fixed cell‐based assay (FCBA) for the detection of myelin oligodendrocyte glycoprotein antibodies (MOG‐IgG) in serum [1].

Leveraging 80 patients from their center with available clinical testing for MOG‐IgG on samples collected on the same day at ARUP Laboratories (Euroimmun FCBA) and Mayo Clinic (LCBA), Vij et al. observed an FCBA sensitivity of 40% and LCBA sensitivity of 100% for MOGAD diagnosis, with comparable specificities (95.7% for both) [1]. These performance estimates are similar to those observed in our study, reinforcing the conclusion that exclusive use of FCBA may result in significant underdiagnosis of MOGAD [2]. Notably, a similar magnitude of discrepancy in the sensitivity of serum MOG‐IgG LCBA and FCBA was also reported by Tea et al. [3] Furthermore, as we discussed in our manuscript, we agree that these findings are not in conflict with the prior comparative study of the ARUP Euroimmun FCBA versus Mayo LCBA assays reported by Smith et al., given that clinical data were unavailable to determine MOGAD diagnosis (precluding the estimation of diagnostic specificity/sensitivity) and the reported good agreement was mainly driven by samples that were negative by both assays (for samples that were positive by at least one assay, agreement was only 70.7% [as calculated by data presented in the manuscript's figures]) [2, 4].

Collectively, these data support that serum MOG‐IgG LCBA should be considered the gold standard assay for the diagnosis of MOGAD.

## Author Contributions

All authors contributed to drafting the work and revising it critically for important intellectual content and provided final approval of this version to be published.

## Conflicts of Interest

Yana Said reports no disclosures. Eoin P. Flanagan has served on advisory boards for Alexion, Genentech, Horizon Therapeutics, and UCB. He has received research support from UCB. He received royalties from UpToDate. Dr. Flanagan is a site principal investigator in a randomized clinical trial of Rozanolixizumab for relapsing myelin oligodendrocyte glycoprotein antibody‐associated disease run by UCB. Dr. Flanagan is a site principal investigator and a member of the steering committee for a clinical trial of satralizumab for relapsing myelin oligodendrocyte glycoprotein antibody‐associated disease run by Roche/Genentech. Dr. Flanagan has received funding from the NIH (R01NS113828). Dr. Flanagan is a member of the medical advisory board of the MOG project. Dr. Flanagan is an editorial board member of Neurology, Neuroimmunology, and Neuroinflammation, the Journal of the Neurological Sciences, and Neuroimmunology Reports. A patent has been submitted on DACH1‐IgG as a biomarker of paraneoplastic autoimmunity. Elias S. Sotirchos has consulted for Alexion, Horizon Therapeutics, Amgen, TG Therapeutics, and Roche/Genentech, and is a site principal investigator for clinical trials funded by Roche/Genentech and UCB.
